# Pulmonary Embolism in Acute Ischaemic Stroke: Evolving Evidence, Diagnostic Challenges, and a Novel Thromboinflammatory Axis Hypothesis

**DOI:** 10.3390/ijms26146733

**Published:** 2025-07-14

**Authors:** Darryl Chen, Sonu M. M. Bhaskar

**Affiliations:** 1 Global Health Neurology Lab, Sydney, NSW 2150, Australia; 2 UNSW Medicine and Health, University of New South Wales (UNSW), South West Sydney Clinical Campuses, Sydney, NSW 2170, Australia; 3 NSW Brain Clot Bank, NSW Health Pathology, Sydney, NSW 2170, Australia; 4 Ingham Institute for Applied Medical Research, Clinical Sciences Stream, Liverpool, NSW 2170, Australia; 5 Department of Neurology & Neurophysiology, Liverpool Hospital and South West Sydney Local Health District, Liverpool, NSW 2150, Australia; 6 Department of Neurology, Division of Cerebrovascular Medicine and Neurology, National Cerebral and Cardiovascular Center (NCVC), Suita 564-8565, Osaka, Japan

**Keywords:** pulmonary embolism, stroke, risk stratification, anticoagulation, prophylaxis

## Abstract

Pulmonary embolism (PE) is an under-recognised yet serious complication in patients with acute ischaemic stroke (AIS), contributing significantly to morbidity and mortality. The interplay of traditional risk factors—such as immobility, endothelial dysfunction, and hypercoagulability—with AIS-specific conditions, including atrial fibrillation, malignancy, and reperfusion therapies, complicates both diagnosis and management. Despite available prophylactic strategies, including low-molecular-weight heparin and intermittent pneumatic compression, their use remains limited by bleeding concerns and a lack of tailored guidelines. This review synthesises the current evidence on the incidence, risk factors, pathophysiology, diagnostic approaches, and preventive strategies for PE in AIS, identifying critical gaps in risk stratification and clinical decision-making. We propose a novel mechanistic framework—*the Brain–Lung Thromboinflammatory Axis Hypothesis*—which posits that stroke-induced systemic inflammation, neutrophil extracellular trap (NET) formation, and pulmonary endothelial activation may drive in situ pulmonary thrombosis independent of deep vein thrombosis. This conceptual model highlights new diagnostic and therapeutic targets and underscores the need for stroke-specific VTE risk calculators, biomarker-guided prophylaxis, and prospective trials to optimise prevention and outcomes in this vulnerable population.

## 1. Introduction

Acute ischemic stroke (AIS) remains the second leading cause of mortality and a major contributor to long-term disability worldwide, highlighting its significant burden on global health systems [1]. Pulmonary embolism (PE), a critical manifestation of venous thromboembolism (VTE), is the third leading cause of cardiovascular mortality globally. When it occurs in the post-stroke setting, it can result in severe morbidity or death. In AIS patients, PE is a clinically significant yet frequently under-recognised complication, affecting approximately 1% of patients within the first 14 days following stroke onset [2]. In the absence of prophylaxis, the risk rises sharply: up to 75% of hemiplegic AIS patients may develop deep vein thrombosis (DVT), of which nearly 20% can progress to PE [3]. Notably, PE is implicated in 25–50% of deaths among AIS patients [4,5], underscoring its clinical and prognostic consequences.

Managing PE in AIS is uniquely challenging due to conflicting priorities in acute care. While anticoagulation remains the cornerstone of PE treatment [6], its use in the early phase of AIS is limited by the elevated risk of haemorrhagic transformation [7]. This complexity is amplified by the heterogeneity of stroke patients, many of whom are elderly, have severe neurological impairments, or live with comorbid conditions such as atrial fibrillation, cancer, or prolonged immobility [8]. Yet, clinical guidelines remain inconsistent, and validated risk stratification tools tailored specifically to AIS populations are conspicuously absent.

Despite advancements in stroke and thromboembolic care, significant knowledge gaps persist in our ability to accurately identify high-risk individuals and deliver effective, individualised prophylaxis. Outcomes remain poor: over 41% of AIS patients who develop PE experience severe disability or death [9]. Emerging evidence suggests that additional risk-modifying factors, including reperfusion therapies such as intravenous thrombolysis (IVT) and endovascular thrombectomy (EVT) [10,11], elevated inflammatory biomarkers [12], and paradoxical embolism via patent foramen ovale (PFO) [13], may further modulate the PE risk. However, these factors remain insufficiently characterised in the current literature, limiting their integration into routine clinical practice.

This review aims to synthesise and critically evaluate the current evidence on the PE incidence, risk factors, diagnostic challenges, and prophylactic strategies in AIS patients. In doing so, it highlights key limitations in existing research and outlines directions for future clinical and translational studies to improve prevention and management in this high-risk population.

## 2. Risk Factors and Pathophysiology

A mechanistic understanding of the factors that predispose people to PE is fundamental to shaping effective prophylactic and therapeutic strategies. These factors include not only traditional elements of VTE risk but also stroke-specific pathophysiological dynamics.

### 2.1. Pathophysiology of Pulmonary Embolism

**Figure 1:**
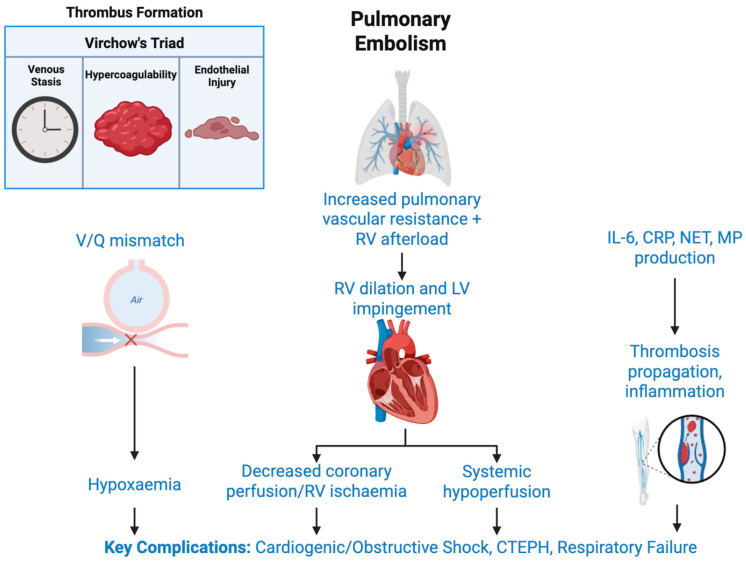
Risk factors and pathophysiology of PE. Abbreviations: CRP, C-reactive protein; CTEPH, chronic thromboembolic pulmonary hypertension; IL-6, interleukin-6; LV, left ventricle; MP, microparticles; NET, neutrophil extracellular trap; RV, right ventricle; V/Q, ventilation/perfusion. This figure was created using BioRender.

The classical model of the PE pathogenesis is grounded in *Virchow’s Triad*—venous stasis, endothelial injury, and hypercoagulability [14]. In AIS patients, each element is accentuated: prolonged immobility leads to venous stasis, cerebral ischaemia provokes systemic inflammation and endothelial activation [15], and the neuroinflammatory cascade fosters a hypercoagulable state (Figure 1) [12,16].

At the molecular level, PE is driven by an orchestrated interaction of prothrombotic mediators [17]. The tissue factor, thrombin, and fibrin are central to clot propagation, while platelet-derived microparticles amplify thrombin generation and inflammatory signalling [18,19]. Recent insights into immunothrombosis, particularly the role of neutrophil extracellular traps (NETs) [20], underscore a link between innate immune activation and thrombus stability, especially in cancer-associated VTE [21]. Biomarkers such as C-reactive protein (CRP) and interleukin-6 (IL-6) are not only correlates of systemic inflammation, but also potential prognostic indicators of thromboembolic risk [12].

If left unresolved, acute thrombi can evolve into chronic thromboembolic pulmonary hypertension (CTEPH), contributing to long-term cardiorespiratory impairment [22]. Early risk stratification is therefore essential. Although scoring systems such as the Wells and Geneva scores offer a starting point, their applicability in AIS populations is limited. Innovations in artificial intelligence and biomarker-based modelling offer opportunities to personalise risk prediction and improve diagnostic accuracy [23].

The aetiology of PE in AIS is multifactorial. Stroke-related immobility, advanced age, cancer, atrial fibrillation, and inherited thrombophilia are well-established contributors [24]. However, stroke-specific factors, such as the extent of the neurological impairment, inflammatory response, and reperfusion therapies, interact with systemic vulnerabilities to shape individual risk profiles [8].

### 2.2. Risk Factors

The risk of PE in AIS arises from an interplay of stroke-related immobility, prothrombotic systemic changes, and predisposing cardiovascular or structural anomalies [8]. Next, we synthesise key clinical and mechanistic risk factors that underpin PE development in this population.

#### 2.2.1. Immobility

Prolonged immobility following AIS, often due to hemiparesis, motor impairment, or reduced consciousness, contributes to venous stasis [25], a central component of Virchow’s triad. Stroke-induced immobility reduces skeletal muscle pump activity, particularly in the lower limbs, increasing the risk of DVT [26] and, subsequently, PE. Observational studies suggest that up to 75% of hemiplegic AIS patients may develop DVT without prophylaxis, with a significant proportion progressing to PE [27]. Stroke severity, as reflected by higher NIH Stroke Scale (NIHSS) scores, correlates strongly with the thromboembolic risk [28].

Early mobilisation is a promising intervention. While pilot studies report reduced VTE complications with early ambulation post-stroke, the evidence remains inconclusive regarding the optimal timing and intensity [29,30]. The AVERT trial, the largest to date, found that very early mobilisation (within 24 h) may be associated with worse outcomes in some subgroups, particularly those with severe strokes [31]. As such, rehabilitation protocols should be individualised, incorporating the stroke severity, functional baseline, and comorbid conditions to maximise benefit while avoiding harm. In addition to physiotherapy, mechanical prophylaxis, such as intermittent pneumatic compression (IPC), may be initiated in the acute phase to reduce the thrombotic risk in patients unable to ambulate early [32].

#### 2.2.2. Hypercoagulability

**Figure 2:**
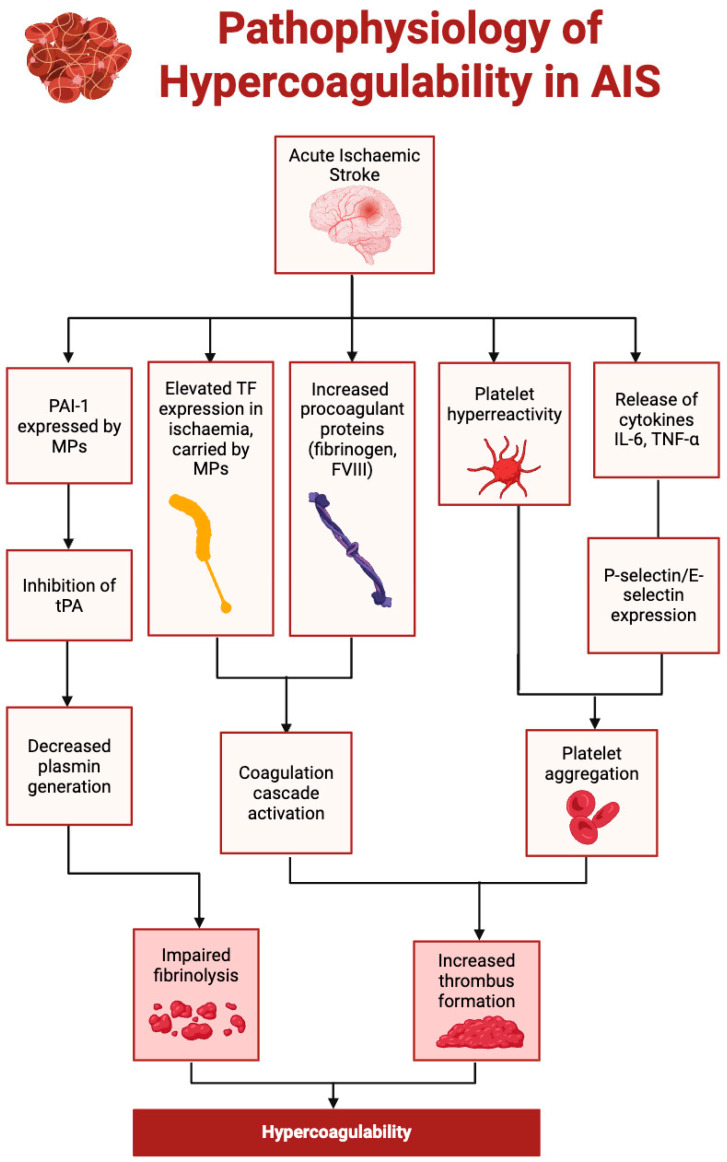
Pathophysiology of hypercoagulability in acute ischemic stroke. Abbreviations: FVIII, clotting factor VIII; IL-6, interleukin-6; TF, tissue factor; TNF-α, tumour necrosis factor alpha; tPA, tissue plasminogen activator; PAI-1, plasminogen activator inhibitor-1; and MP, microparticles. This figure was created with BioRender.

AIS induces a prothrombotic state through a complex cascade involving inflammation, endothelial injury, coagulation activation, and impaired fibrinolysis [33]. Ischaemic brain injury triggers the release of pro-inflammatory cytokines, such as interleukin-6 (IL-6) and tumour necrosis factor-alpha (TNF-α) [34], which upregulate adhesion molecules (e.g., P-selectin, E-selectin) on endothelial cells, promote platelet aggregation, and amplify thrombin generation (Figure 2) [35,36].

**Figure 3:**
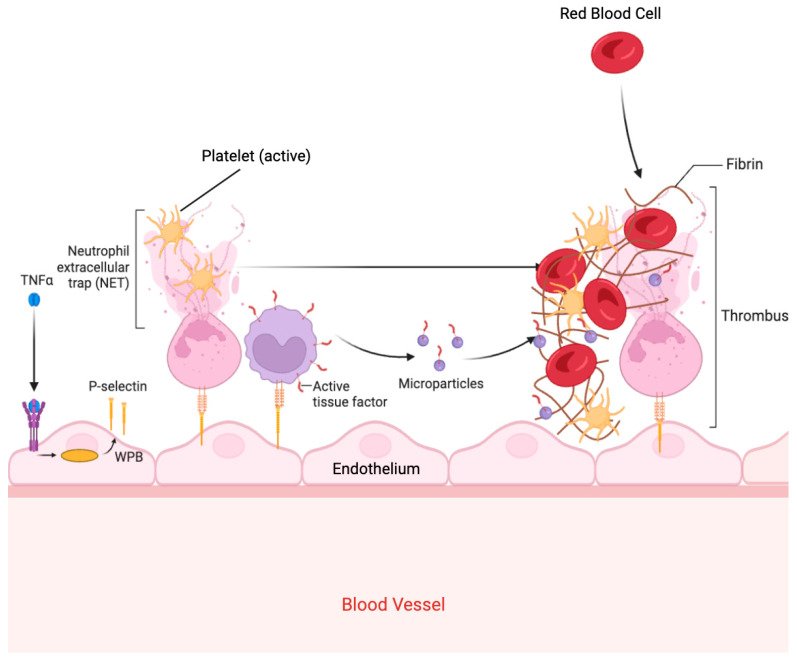
Thrombus formation in hypercoagulability. Abbreviations: TNF-α, Tumour Necrosis Factor Alpha; WPB, Weibel Palade Bodies. This figure was created with BioRender.

In parallel, AIS leads to increased circulating levels of fibrinogen, factor VIII, and microparticle-associated tissue factor, accelerating the activation of the extrinsic coagulation pathway [37]. This environment favours both arterial and venous thrombosis. Importantly, AIS also disrupts endogenous fibrinolysis via the upregulation of plasminogen activator inhibitor-1 (PAI-1), which inhibits the tissue plasminogen activator (tPA) and impedes clot resolution (Figure 3) [38]. The resulting imbalance between coagulation and fibrinolysis promotes clot persistence and propagation [39,40,41].

The hypercoagulable state in AIS may be exacerbated by coexisting factors such as active malignancy or inherited thrombophilia [42], both of which independently heighten the thrombotic risk [43,44]. These patients represent a particularly vulnerable subgroup requiring tailored prophylactic strategies.

While inflammatory biomarkers—including IL-6, C-reactive protein (CRP), and TNF-α—are frequently elevated in both AIS and PE, their predictive value for PE development remains limited when used in isolation [45,46,47,48,49]. There is a growing interest in the development of integrated risk algorithms that combine biomarker data with clinical and imaging parameters to improve risk stratification and guide prophylactic decision-making.

#### 2.2.3. Patent Foramen Ovale

Patent foramen ovale (PFO) is a congenital interatrial communication present in approximately 20–25% of the general population and identified in nearly 50% of patients with cryptogenic stroke [50]. Its role in stroke pathogenesis is increasingly recognised, particularly through the mechanism of paradoxical embolism—the transit of venous thrombi into the systemic circulation via a right-to-left atrial shunt, bypassing the pulmonary filter [51].

Under normal haemodynamic conditions, the left atrial pressure exceeds the right, keeping the foramen functionally closed. However, transient or sustained elevations in the right atrial pressure, such as those induced by Valsalva manoeuvres (coughing, sneezing, straining), pulmonary hypertension, or acute pulmonary embolism, can reverse this pressure gradient, facilitating embolic crossover [52].

In AIS patients with concomitant PE, right heart strain or elevated pulmonary pressures may further increase the likelihood of PFO-mediated embolic transit [53]. This interplay complicates both diagnostic and therapeutic pathways, especially when clinical suspicion arises for dual embolic pathologies. Additionally, patients with PFO and atrial septal aneurysm or a large interatrial shunt demonstrate a higher risk of recurrent embolic events [54].

The management of PFO in the AIS population requires individualised risk–benefit assessments [55]. The closure of PFO has demonstrated benefits in secondary stroke prevention for carefully selected cryptogenic stroke patients with high-risk anatomical features [56]. However, evidence regarding the closure in the setting of active PE or recent thrombolysis is limited, and concerns regarding the procedural risks, residual shunt, or recurrence of PE must be weighed [50,57,58].

The current consensus favours anticoagulation in the acute phase, with deferred closure considered after a comprehensive stroke workup [59]. The decision to close must be based on a multidisciplinary evaluation, integrating the stroke subtype, embolic burden, PFO morphology, and bleeding risk [60]. Randomised trials specifically addressing closure timing in AIS patients with a concurrent PE are needed.

#### 2.2.4. Atrial Fibrillation

AF is a well-established risk factor in AIS patients, primarily due to its association with the formation of left atrial appendage thrombus [61]. The absence of coordinated atrial contraction results in blood stasis and promotes thrombus development [62], with embolisation to the cerebral circulation as a frequent consequence. AF underpins a substantial proportion of cardioembolic strokes and is a key determinant of stroke severity and recurrence [63].

AF is also implicated in the pathogenesis of PE. Epidemiological data suggest that approximately 12.5% of patients presenting with acute PE have coexisting AF [64], indicating shared thromboembolic mechanisms and systemic prothrombotic states. In AIS patients, the concurrence of AF and PE signifies a particularly high-risk phenotype requiring careful therapeutic navigation [62].

Management becomes complex when both conditions co-occur, as anticoagulation—central to preventing further thromboembolic events—must be judiciously timed. The risk of a haemorrhagic transformation is elevated in AIS, particularly following reperfusion therapies such as IVT or EVT [7]. Current guidelines recommend delaying the anticoagulation initiation for at least 24 h post-reperfusion, with a confirmation of haemorrhage exclusion on neuroimaging.

AF may further exacerbate the right atrial pressure [65], particularly in the context of underlying pulmonary hypertension or ventricular dysfunction, potentially increasing the right-to-left shunt in patients with PFO. This creates a plausible pathway for a paradoxical embolism [51], linking the AF, PFO, and stroke in a high-risk triad. In such cases, a multidisciplinary strategy—integrating cardiology, neurology, and haematology—is essential to tailor anticoagulation regimens and consider adjunctive interventions such as PFO closure or rhythm control.

#### 2.2.5. Malignancy

Malignancy is a well-established risk factor for VTE, including PE, and plays a significant yet underappreciated role in AIS patients [24,43,44]. Cancer induces a hypercoagulable state through multiple mechanisms: the tumour cell expression of tissue factor [66], secretion of procoagulant microparticles, and inflammatory cytokine release. Furthermore, chemotherapy, radiotherapy, central venous catheters, and surgical interventions amplify thrombotic risk.

The phenomenon of cancer-associated stroke (CAS) is increasingly recognised and often presents with cryptogenic features and concurrent PE or DVT [44]. Up to 10–15% of patients with ischemic stroke have an underlying occult malignancy, with higher risks observed in adenocarcinomas, particularly of the pancreas, lung, stomach, and prostate [67]. Elevated D-dimer, CRP, and fibrinogen levels are common in this subgroup and correlate with poor outcomes [68]. Studies demonstrate that CAS is linked with an elevated tissue factor expression and microthrombi formation [43,44,69], while others describe a higher PE recurrence in cancer patients despite standard anticoagulation [70,71,72,73].

The presence of cancer alters the risk–benefit balance of anticoagulation, particularly in AIS patients undergoing reperfusion therapy [74]. Low-molecular-weight heparin (LMWH) remains the standard for VTE prophylaxis in cancer [75,76], though direct oral anticoagulants (DOACs) have emerged as alternatives in selected patients [77]. Stratification tools incorporating cancer-related variables may aid in identifying AIS patients requiring early screening for an occult malignancy or enhanced thromboprophylaxis [78,79,80].

#### 2.2.6. Obesity and Metabolic Syndrome

Obesity is an independent risk factor for both AIS and VTE [81]. Excess adiposity is associated with systemic inflammation, impaired fibrinolysis, endothelial dysfunction, and elevated levels of prothrombotic factors, such as plasminogen activator inhibitor-1 (PAI-1), fibrinogen, and factor VII [82,83]. The adipose tissue secretes pro-inflammatory adipokines (e.g., leptin, resistin) and cytokines (e.g., IL-6, TNF-α) that perpetuate a prothrombotic state [84].

Obese patients post-stroke may experience prolonged immobility due to a reduced baseline physical capacity, sarcopenia, or mobility-related complications, further increasing the risk of VTE [85]. Moreover, central obesity, a hallmark of metabolic syndrome, is associated with increased platelet reactivity and thrombin generation [86]. In AIS populations, obesity paradoxically correlates with slightly better early survival but worse long-term functional outcomes, partly due to the higher PE incidence [87,88]. Obese patients with metabolic syndrome require tailored VTE prophylaxis strategies, including dose-adjusted anticoagulation and early mobilisation protocols that account for biomechanical limitations [89]. Standard prophylaxis may be suboptimal in obese individuals [90]; the efficacy of mechanical methods like intermittent pneumatic compression (IPC) may be diminished due to a reduced calf compression effectiveness, necessitating a pharmacological intervention [91,92].

#### 2.2.7. Sex-Specific Risk

Sex-based differences in the PE risk and outcomes after AIS warrant greater attention [93]. Women may have a higher risk of VTE post-stroke due to hormonal influences, the use of hormone replacement therapy, and differences in platelet reactivity and endothelial function [94]. Conversely, men often exhibit higher baseline D-dimer levels and increased thrombotic burdens in acute PE, potentially confounding diagnostic interpretation [95]. The bleeding risk also differs by sex, with older women at increased risk of haemorrhagic complications from anticoagulation [96,97]. These disparities underscore the need for sex-specific risk stratification models and anticoagulation protocols in AIS patients with suspected or confirmed PE [98,99].

#### 2.2.8. Inherited and Acquired Thrombophilia

Thrombophilias, both inherited and acquired, represent an important yet often overlooked risk domain in AIS patients at risk of PE [100]. Common heritable conditions include the Factor V Leiden mutation, the prothrombin G20210A mutation, protein C or S deficiency, and antithrombin III deficiency [101]. These disorders increase the risk of unprovoked DVT and PE and may be particularly relevant in younger AIS patients with cryptogenic features [102].

Acquired thrombophilias, such as antiphospholipid syndrome (APS), have been implicated in both arterial and venous thrombotic events [103]. APS is characterised by circulating antiphospholipid antibodies (e.g., lupus anticoagulant, anticardiolipin antibodies), which activate endothelial cells and platelets, leading to recurrent thrombosis [104]. Importantly, APS-associated strokes may co-occur with PE, necessitating long-term anticoagulation [105]. Warfarin remains preferred over DOACs in high-risk thrombophilias such as APS, given the increased rate of recurrent events with the DOAC use in these patients [106].

Routine thrombophilia screening is not recommended for all AIS patients but should be considered [107] in those with (a) unexplained or recurrent thrombotic events; (b) a family history of thrombosis; (c) a stroke at a young age (<50 years) with no identifiable risk factors; and (d) concomitant PE or DVT in the absence of triggering factors.

### 2.3. Additional Consideration in Young Stroke and Emerging Risk Populations

Young patients with cryptogenic stroke represent a unique population where the PE risk may be underestimated [108]. In this group, undiagnosed thrombophilias, autoimmune disorders (e.g., antiphospholipid syndrome, systemic lupus erythematosus), and post-COVID prothrombotic states are increasingly recognised contributors to both arterial and venous thromboembolism [109,110]. COVID-19-associated coagulopathy has been linked to elevated NETs, endothelial dysfunction, and persistent hyperinflammatory responses, which may heighten the PE risk even in the absence of traditional risk factors [111]. Incorporating screening for the autoimmune markers and recent infection history may help identify vulnerable individuals requiring early prophylaxis and a long-term follow-up.

## 3. Diagnostics

Diagnosing PE in patients with AIS presents substantial clinical challenges. Overlapping features, such as dyspnoea, hypoxia, tachypnoea, and tachycardia, may be misattributed to neurological impairment, aspiration pneumonia, or deconditioning [5]. The primary focus during AIS management is typically neurological recovery, which may delay or obscure the recognition of cardiopulmonary complications [5]. Moreover, common post-stroke sequelae—including impaired communication (e.g., dysphasia, cognitive dysfunction) and reduced consciousness—limit accurate symptom reporting [112]. PE may also be erroneously diagnosed as pneumonia, particularly given that a fever is present in up to two-thirds of PE cases [113].

These diagnostic ambiguities are compounded by immobility and systemic inflammation, which increase the baseline risk for VTE in the AIS population [25]. Given these complexities, the timely and accurate diagnosis of PE in AIS requires a multimodal approach that integrates clinical suspicion, biomarker profiles, and imaging, each with context-specific limitations (Table 1). However, stroke-specific diagnostic pathways remain underdeveloped, and no guidelines currently provide an AIS-tailored diagnostic algorithm.

### 3.1. Diagnostic and Management Algorithm

**Figure 4:**
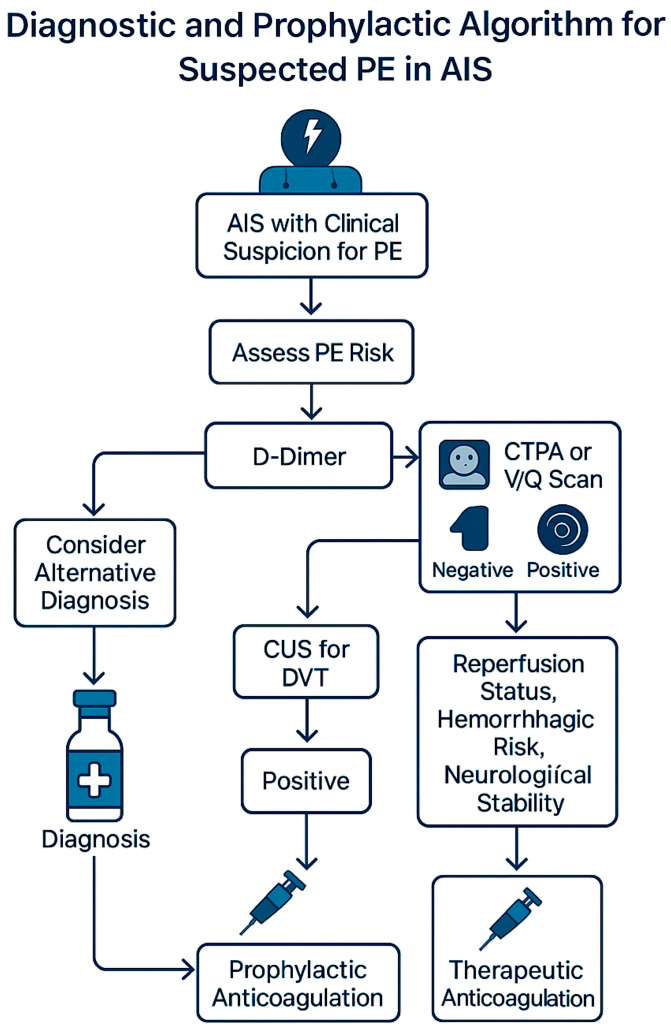
A diagnostic and prophylactic algorithm for suspected pulmonary embolism in acute ischemic stroke. A stepwise approach for assessing the PE risk in AIS patients, integrating the D-dimer interpretation, imaging selection (CTPA, V/Q scan, CUS), and timing of prophylactic or therapeutic anticoagulation based on the reperfusion status, haemorrhagic risk, and neurological stability.

Given the overlapping clinical features and high morbidity associated with PE in AIS, a structured diagnostic and prophylactic approach is essential. Figure 4 outlines a practical, stepwise algorithm integrating clinical suspicion, biomarker thresholds, and imaging modalities to guide diagnosis and management in AIS patients suspected of PE.

### 3.2. D-Dimer

D-dimer is a fibrin degradation product widely used to rule out PE in low-risk patients. It has a high sensitivity but a poor specificity, particularly in inflammatory and hypercoagulable states such as AIS [114]. Stroke-induced tissue damage and systemic inflammation can independently elevate D-dimer, limiting its diagnostic precision in this population [115,116]. Nevertheless, adjusting D-dimer thresholds based on the clinical pretest probability (e.g., age-adjusted or Wells-score–adjusted cut-offs) can improve the specificity without compromising the negative predictive value [117]. While a normal D-dimer may be useful to exclude PE in select AIS patients with low clinical suspicion, elevated levels should be interpreted cautiously and always in conjunction with clinical and radiological findings.

### 3.3. CT Pulmonary Angiography

CT Pulmonary Angiography (CTPA) remains the gold standard for a definitive PE diagnosis due to its high sensitivity and specificity and its capacity to directly visualise intraluminal thrombi in pulmonary arteries. However, its utility in AIS is often limited by logistical and clinical barriers, particularly in patients with severe neurological deficits, haemodynamic instability, or contraindications to the iodinated contrast (e.g., renal dysfunction, allergy). In such patients, a risk–benefit analysis is essential. Whenever feasible, CTPA should be pursued in AIS patients with a high clinical suspicion of PE, especially those with unexplained hypoxia, hypotension, or right heart strain on echocardiography [118].

### 3.4. Ventilation Perfusion (V/Q) Scan

The V/Q scan provides an alternative imaging modality for patients in whom CTPA is contraindicated [119]. It is particularly advantageous in patients with renal impairment, pregnancy, or a contrast media allergy. However, its diagnostic yield is reduced in patients with a pre-existing pulmonary disease, a poor respiratory effort, or non-specific ventilation abnormalities—conditions that are common in AIS [120]. Low-probability or indeterminate V/Q scan results necessitate further investigation, and interpretation requires an integration with clinical probability scores (e.g., modified Wells or revised Geneva scores) [23].

### 3.5. Compression Ultrasonography

CUS is a non-invasive, bedside-accessible modality used to detect DVT, which is a frequent antecedent of PE. While not diagnostic of PE per se, the presence of DVT in an AIS patient, especially in the setting of hypoxia or tachycardia, strongly suggests a thromboembolic source [16]. Given its safety, cost-effectiveness, and ease of application, CUS is a first-line adjunctive tool, particularly in patients with an impaired cardiopulmonary reserve where advanced imaging is not feasible [16].

### 3.6. Echocardiography

Echocardiography, especially transthoracic (TTE) or transoesophageal (TEE), can assess right ventricular (RV) strain, an indirect marker of PE [121]. Signs such as RV dilatation, interventricular septal flattening, and a reduced tricuspid annular plane systolic excursion (TAPSE) may support a presumptive diagnosis in unstable patients [16]. Biomarkers such as troponin and B-type natriuretic peptide (BNP) may also reflect RV strain but are non-specific and should be interpreted in context [122]. Echocardiography is particularly useful in guiding emergent decisions, such as thrombolysis or catheter-based interventions, in haemodynamically unstable AIS patients with suspected PE [123].

## 4. Management

The management of PE in AIS settings presents a unique clinical dilemma, requiring the careful navigation between preventing thromboembolic events and avoiding haemorrhagic transformation. This challenge is compounded by the lack of AIS-specific guidelines, inconsistent use of prophylaxis, and limited validated risk stratification tools tailored to stroke populations.

### 4.1. Risk Stratification and Diagnosis

The early identification of AIS patients at a high risk of PE is essential. General VTE risk tools such as the Wells, Geneva, and Caprini scores were developed for non-neurological cohorts and inadequately capture AIS-specific factors such as neurological deficits, prolonged immobility, and stroke severity. Consequently, their utility in guiding prophylaxis or acute interventions is limited [124,125]. Future risk prediction models should integrate stroke-specific variables—such as the NIHSS, infarct location and size, degree of immobility, and inflammatory markers (e.g., IL-6, D-dimer)—with imaging findings to generate more accurate, individualised assessments.

Emerging artificial intelligence (AI) tools, including machine learning algorithms trained on electronic health records and imaging data, have shown promise in stratifying the PE risk among AIS patients [126]. For instance, convolutional neural networks (CNNs) can identify subclinical right ventricular strain on echocardiography, while natural language processing (NLP) may automate the recognition of early clinical signs from documentation. Integrating AI models with biomarker panels (e.g., D-dimer, IL-6) may enhance the diagnostic accuracy and reduce the time to intervention.

While CTPA remains the diagnostic gold standard for PE, its application must be weighed against renal function, hemodynamic stability, and transport logistics in critically ill AIS patients [127]. Biomarkers like D-dimer, though useful as initial screening tools, suffer from a low specificity in stroke due to baseline elevations from inflammation and tissue injury. A tiered diagnostic approach combining a clinical assessment, D-dimer thresholds, and selective imaging is prudent [116,117,118].

### 4.2. Prophylactic Management

Table 2 summarises current management guidelines for PE prophylaxis in AIS patients, comparing pharmacological and mechanical approaches while highlighting their respective benefits and limitations.

#### 4.2.1. Pharmacological Prophylaxis

Pharmacological prophylaxis with low-molecular-weight heparin (LMWH) or unfractionated heparin (UFH) is recommended in immobile AIS patients to reduce the risk of VTE [131]. LMWH is preferred due to its superior efficacy and once-daily dosing. However, anticoagulation should be delayed for at least 24 h post-stroke onset and only initiated after confirming the absence of haemorrhages on neuroimaging [132].

Despite guideline endorsement, anticoagulation remains underutilised, primarily due to concerns about bleeding, especially in elderly patients or those undergoing reperfusion therapy. Further studies are needed to refine the timing, dosage, and safety thresholds, particularly in patients with a high baseline bleeding risk [133]. Direct oral anticoagulants (DOACs) are increasingly used in AIS secondary to atrial fibrillation [134], but their role in primary PE prophylaxis post-stroke remains under-investigated. Ongoing trials may clarify their safety profile in early AIS settings [135,136].

#### 4.2.2. Mechanical Prophylaxis

Intermittent pneumatic compression (IPC) is the most effective non-pharmacological prophylactic strategy in AIS, especially in patients with contraindications to anticoagulation [137]. The CLOTS 3 trial showed a significant reduction in the DVT incidence when IPC was initiated within 72 h of the stroke onset [138]. The early use of IPC may be particularly valuable in the post-thrombolysis period, where anticoagulation is deferred [139,140].

Graduated compression stockings (GCS) are no longer recommended due to their limited efficacy and risks of skin breakdown [9]. Neuromuscular electrical stimulation (NMES), though mechanistically appealing, lacks conclusive evidence for PE prevention and should be considered investigational [141].

#### 4.2.3. Prophylaxis in the Context of Reperfusion Therapy

Patients undergoing IVT or EVT pose a distinct risk profile [142]. Procedures involving prolonged recanalisation times and endovascular manipulation may contribute to endothelial activation and a prothrombotic state. Retrospective data suggest an increased incidence of DVT in EVT-treated patients [10], although robust prospective studies are lacking.

In this population

- Anticoagulation should be delayed for at least 24 h after IVT or EVT [143,144];
- Neuroimaging is mandatory before initiating prophylactic heparin to exclude haemorrhagic transformation [59,145];
- Sequential management—reperfusion first, followed by delayed anticoagulation—is preferred over simultaneous thrombolysis for PE [10].

Patients with atrial fibrillation and those with renal impairment require individualised decisions regarding anticoagulation initiation and dosing. For AF, long-term anticoagulation is typically warranted [146], but a delayed initiation may be necessary to reduce the early bleeding risk [143]. Renal function should guide the selection between LMWH and DOACs.

### 4.3. Acute Management

The acute management of PE in patients with AIS presents a therapeutic conflict: while PE mandates urgent anticoagulation to prevent cardiovascular collapse, AIS, particularly post-reperfusion, poses a high risk of haemorrhagic transformation. The rarity of this clinical overlap and the absence of standardised protocols demand a case-by-case, multidisciplinary approach.

UFH remains the treatment of choice for acute PE due to its rapid onset, short half-life, and ease of reversal. Timely initiation is crucial, as delays beyond 24 h are associated with increased mortality [6]. However, in AIS patients, especially those who have undergone IVT or EVT, anticoagulation within the first 24 h should be deferred unless the haemorrhagic risk is minimal and confirmed by follow-up neuroimaging [7].

In patients with contraindications to anticoagulation or those presenting with massive PE and haemodynamic instability, mechanical interventions such as catheter-directed thrombectomy (CDT) or surgical embolectomy may be considered. CDT offers the advantage of a targeted thrombus removal with a lower systemic bleeding risk and has shown promise in reducing in-hospital mortality. However, large-scale, stroke-specific randomised controlled trials are lacking [147]. In AIS patients with contraindications to anticoagulation and evidence of DVT or atrial/ventricular thrombus (AVC), temporary inferior vena cava (IVC) filters may be considered to prevent pulmonary embolism. However, current evidence indicates limited long-term benefits, and filter placements should be reserved for carefully selected patients. Where feasible, early removal is recommended to minimise filter-related thrombosis and migration risks.

Importantly, the sequence of therapy should prioritise neurological stabilisation: rapid cerebral reperfusion via EVT in eligible patients, followed by a reassessment of the anticoagulation timing based on the radiographic and clinical evolution [148,149].

### 4.4. Long-Term Management

Long-term management in patients with both acute ischaemic stroke (AIS) and pulmonary embolism (PE) centres around sustained anticoagulation, comprehensive risk factor modifications, and close monitoring for recurrence or complications.

#### 4.4.1. Anticoagulation Strategies

Oral anticoagulation remains the cornerstone of PE treatment, with direct oral anticoagulants (DOACs) such as apixaban or rivaroxaban generally preferred over warfarin due to their ease of use, predictable pharmacokinetics, and favourable safety profile [150,151]. However, in select populations—such as patients with end-stage renal disease, mechanical heart valves, or antiphospholipid syndrome—vitamin K antagonists (VKAs) like warfarin remain the anticoagulant of choice.

The duration of anticoagulation should be individualised. Patients with unprovoked PE or those who experience recurrent thromboembolic events may require extended or indefinite therapy, whereas patients with identifiable and transient risk factors may be safely managed with a shorter course of 3 to 6 months [152]. In the context of AIS, long-term anticoagulation is particularly important for secondary prevention in cardioembolic stroke, such as that associated with atrial fibrillation or left ventricular thrombus [145]. DOACs are typically favoured unless contraindicated by comorbidities or drug interactions [153].

An ongoing follow-up is essential to ensure treatment safety and efficacy. This includes regular international normalised ratio (INR) monitoring for patients on warfarin (with a target INR of 2.0–3.0) [9,154], the periodic assessment of the renal function for patients on DOACs, and clinical and radiological surveillance for signs of recurrent stroke, recurrent PE, or the development of chronic thromboembolic pulmonary hypertension (CTEPH) [23].

#### 4.4.2. Cognitive Sequelae of Subclinical PE

Emerging evidence suggests that undetected or subclinical PE in AIS patients may contribute to persistent hypoxia, exacerbating the post-stroke cognitive decline [155,156,157]. Chronic low-grade hypoxemia can impair neuronal recovery and promote neuroinflammation, particularly in vulnerable brain regions such as the hippocampus [158]. Studies in other hypoxic conditions support this association, but the relationship remains underexplored in stroke populations. Integrating routine oxygenation monitoring and cognitive assessments post-stroke may help uncover silent contributors to poor recovery trajectories [159].

#### 4.4.3. Secondary Prevention and Rehabilitation

Comprehensive secondary prevention in patients with both AIS and PE should prioritise the aggressive management of modifiable vascular risk factors [160]. This includes the optimal control of hypertension, diabetes, and dyslipidaemia, as well as smoking cessation and the implementation of lifestyle interventions such as weight reduction, regular physical activity, and dietary modifications [62]. These measures not only reduce the risk of recurrent thromboembolic and cerebrovascular events but also contribute to improved overall cardiovascular health [160].

#### 4.4.4. Integrated Neuro-Pulmonary Rehabilitation

Emerging evidence supports the value of integrating pulmonary rehabilitation into standard neurorehabilitation programs for AIS patients with PE [161]. Early, structured pulmonary rehab, including breathing exercises, inspiratory muscle training, and aerobic conditioning, may enhance cardiopulmonary function, reduce dyspnoea, and support functional neurological recovery [162]. This interdisciplinary approach could be particularly beneficial in patients with persistent hypoxia, reduced exercise tolerance, or evolving CTEPH [163]. Future studies should evaluate combined neuro-pulmonary rehab protocols to optimise recovery trajectories and quality of life [164].

In survivors of PE, pulmonary rehabilitation has demonstrated benefits in improving exercise tolerance, reducing dyspnoea, and enhancing quality of life [165]. For patients who continue to experience symptoms of exertional breathlessness or who develop signs of CTEPH, targeted pharmacologic therapies such as endothelin receptor antagonists or phosphodiesterase inhibitors may be warranted [143,166].

Looking ahead, several areas require further exploration. These include the validation of combined long-term anticoagulation strategies tailored for patients with a dual pathology (AIS and PE), research into integrated rehabilitation pathways that address both neurological and pulmonary recovery, and the development of remote monitoring platforms to detect anticoagulation-related complications early [167]. Such innovations have the potential to personalise care, reduce morbidity, and improve long-term outcomes in this complex patient population.

## 5. Discussion

PE is a clinically significant but often under-recognised complication in patients with AIS. Our review highlights key gaps in the literature and clinical practice: (1) the absence of AIS-specific risk stratification tools for VTE; (2) diagnostic ambiguity due to overlapping cardiorespiratory and neurological symptoms; (3) limited evidence guiding the safe use of anticoagulation post-reperfusion therapy; and (4) a lack of standardised guidelines tailored to AIS patients. These limitations hinder the accurate identification, timely diagnosis, and optimal management of PE in this high-risk population.

### 5.1. The Brain–Lung Thromboinflammatory Axis Hypothesis

We introduce a novel hypothesis that explains the occurrence of PE in AIS through a dysregulated brain–lung thromboinflammatory axis, characterised by systemic neutrophil activation, endothelial dysfunction, and impaired alveolar–capillary barrier integrity. We propose that AIS initiates a cascade of systemic inflammation and neurohumoral activation that primes the pulmonary vasculature for thrombosis [168]. Specifically, AIS induces a robust inflammatory cascade involving the activation of the hypothalamic–pituitary–adrenal axis and systemic sympathetic discharge [111]. This response leads to peripheral neutrophil activation and the release of neutrophil extracellular traps (NETs), a phenomenon known as stroke-induced NETosis [169,170]. NETs promote platelet adhesion, fibrin deposition, and intravascular coagulation—systemically, but particularly within low-flow venous beds [171,172].

Simultaneously, circulating cytokines along with elevated catecholamines activate the pulmonary endothelium [173]. Experimental models show that cerebral ischaemia can lead to pulmonary endothelial activation, mediated by circulating cytokines (e.g., IL-6, TNF-α, HMGB1) and catecholamines [174,175,176]. This causes the pulmonary vascular endothelium to upregulate the adhesion molecules (VCAM-1, E-selectin) and tissue factor, creating a prothrombotic environment in the pulmonary vasculature even in the absence of DVT [177,178].

We further hypothesise that this prothrombotic pulmonary environment may lead to in situ pulmonary thrombosis, independent of DVT, resembling mechanisms observed in COVID-19-associated immunothrombosis [111]. The inflammatory priming of the pulmonary vasculature could promote in situ microthrombus formation—a non-embolic PE variant—analogous to what is observed in COVID-19-associated immunothrombosis [179]. In AIS, this may be a result of NETs, platelet activation, and dysfunctional alveolar–capillary exchange [180,181]. Recent studies suggest that AIS leads to the release of brain-derived extracellular vesicles (EVs) carrying procoagulant cargo (e.g., phosphatidylserine, TF-positive microparticles) [182]. These EVs may circulate to the lungs and disrupt the pulmonary vascular homeostasis, promoting clot formation and vascular occlusion [183].

This hypothesis challenges the classical view that PE in AIS solely results from emboli originating in the lower extremities. Instead, it suggests a mechanism of stroke-induced pulmonary thromboinflammation, driven by neurovascular-immune interactions [184]. Clinically, our hypothesis urges the consideration of PE in AIS patients who develop hypoxia without detectable DVT and highlights the potential utility of biomarkers such as citrullinated histones, cell-free DNA, and EV signatures for risk stratification [185]. Finally, it opens new avenues for therapeutic exploration, including NET inhibitors (e.g., DNase, PAD4 inhibitors) and endothelial-targeted strategies, to prevent thromboinflammatory complications in stroke care [186,187].

### 5.2. Pathophysiological Considerations

AIS triggers a systemic prothrombotic state by simultaneously engaging all components of Virchow’s triad: venous stasis from immobility, endothelial dysfunction due to inflammatory cytokines, and hypercoagulability mediated by the upregulated tissue factor and impaired fibrinolysis. The platelet hyperactivity and NET formation further stabilise thrombi. While the PFO-related paradoxical embolism remains a recognised contributor to PE in cryptogenic stroke, newer inflammatory and immunothrombotic mechanisms provide a broader, more inclusive framework for understanding the PE risk in AIS [35,36,53].

### 5.3. Diagnostic and Management Challenges

Diagnosing PE in AIS patients remains challenging. Common symptoms of PE—hypoxia, tachypnoea, and dyspnoea—overlap with neurological deficits or post-stroke pneumonia, and communication limitations may further obscure clinical presentations [111]. D-dimer, though sensitive, lacks specificity in AIS due to stroke-induced inflammation [116]. While CTPA is the gold standard [118], it may be contraindicated or impractical in critically ill stroke patients [23]. V/Q scans and compression ultrasonography (CUS) offer alternatives but are often indirect. Stroke-specific diagnostic algorithms are lacking, and current tools like the Wells and Geneva scores fail to incorporate neurological factors such as stroke severity or immobility. Future improvements should include validated AIS-specific PE risk calculators, biomarker-driven triage algorithms (e.g., IL-6, D-dimer), and the integration of point-of-care ultrasound (POCUS) and machine learning approaches into clinical workflows [126].

The management of PE in AIS demands a careful balance between preventing thromboembolism and avoiding haemorrhagic transformation. In immobile patients, LMWH or UFH are commonly used, with LMWH preferred for its efficacy and convenience [27]. However, anticoagulation should be delayed at least 24 h after reperfusion therapy and only initiated following confirmatory imaging. Intermittent IPC, supported by CLOTS 3 trial data [139,140], remains the cornerstone of mechanical prophylaxis, particularly in patients at a high bleeding risk. GCS are discouraged due to poor efficacy. In cases of coexistent AIS and high-risk or massive PE, catheter-directed thrombectomy (CDT) may be considered in patients who are not anticoagulation candidates [188]. Sequential therapy, AIS reperfusion followed by delayed anticoagulation, minimises the haemorrhagic risk [189]. Long-term management includes sustained anticoagulation (preferably DOACs unless contraindicated), risk factor modification, and monitoring for recurrent events or complications such as CTEPH [23].

### 5.4. Future Directions

To improve outcomes, future research must address several unmet needs:

I. The development of AIS-specific VTE risk calculators that integrate the NIHSS, infarct subtype, immobility, and biomarkers to guide prophylaxis.
II. Randomised trials evaluating the safety and efficacy of DOACs in early post-stroke prophylaxis, particularly after IVT or EVT.
III. Biomarker-guided anticoagulation protocols, using serial measurements of IL-6, D-dimer, and potentially NET-related markers, to personalise therapy and determine the timing of anticoagulation.
IV. Emerging therapeutic avenues include targeting NETs and endothelial activation [186]. Preclinical studies have shown that PAD4 inhibitors and DNase I can reduce NET-mediated thrombosis and improve outcomes in thromboinflammatory conditions. Similarly, agents that stabilise endothelial function, such as recombinant thrombomodulin, statins, and sphingosine-1-phosphate analogues, may mitigate endothelial injury and hyperpermeability in the pulmonary vasculature [190]. Anti-cytokine therapies (e.g., IL-6 or TNF-α inhibitors) may also hold promise in modulating the systemic inflammatory response post-stroke [111]. These strategies warrant an investigation in AIS populations, particularly in those at high risk for in situ pulmonary thrombosis.
V. An investigation of combined strategies, such as early IPC with low-dose anticoagulation, to optimise safety and efficacy, especially in patients with elevated bleeding risk [91,92,191].
VI. The validation of stroke–PE rehabilitation models and remote monitoring platforms for the early detection of anticoagulation-related complications and functional recovery.

Significant global disparities exist in the diagnosis and prevention of PE in AIS. In low- and middle-income countries (LMICs), limited access to CT Pulmonary Angiography, D-dimer testing, and compression ultrasonography hampers timely diagnosis [192,193]. Additionally, cost and resource constraints often restrict the use of IPC and pharmacological prophylaxis [191]. In contrast, high-income countries benefit from protocolised care pathways and advanced imaging access [23]. These inequalities underscore the need for context-specific, resource-adapted protocols and point-of-care innovations to bridge diagnostic and therapeutic gaps in stroke care globally [194].

## 6. Conclusions

PE is a clinically significant yet under-recognised complication in patients with AIS [23,46,118]. Our review underscores the multifactorial nature of PE in this population, encompassing traditional thromboembolic risks, such as immobility and hypercoagulability, alongside emerging contributors including cancer [24], atrial fibrillation [62], and paradoxical embolism [51]. We propose a novel pathophysiological framework—the *Brain–Lung Thromboinflammatory Axis Hypothesis*—which highlights how stroke-induced systemic inflammation and endothelial activation may drive pulmonary thrombosis, even in the absence of deep vein thrombosis. Despite the availability of pharmacological and mechanical prophylactic strategies, their application remains inconsistent, largely due to the lack of AIS-specific diagnostic algorithms and validated risk prediction tools. Current diagnostic practices and treatment protocols, often extrapolated from non-stroke populations, fail to address the unique clinical and haemodynamic challenges presented by AIS [195]. To improve outcomes, we advocate for the development of stroke-specific VTE risk calculators, biomarker-guided anticoagulation protocols, and prospective trials evaluating the safety and efficacy of DOACs in the early post-stroke setting. Future research should also explore combined mechanical and pharmacologic strategies, as well as novel therapeutic targets such as NET inhibition and endothelial stabilisation [169,186]. These innovations are essential to optimise prevention, refine diagnoses, and personalise the management of PE in AIS.

## Figures and Tables

**Table 1:** Diagnostic modalities of PE in AIS patients.

Modality	Utility	Limitations in AIS patients
D-dimer [51,52]	High sensitivity for ruling out PE in low-risk patients Useful when combined with pretest probability	Low specificity in AIS due to inflammation and tissue damage Elevated baseline levels can confound interpretation
CTPA [50]	Gold standard for visualising pulmonary thrombi High sensitivity and specificity	It may be unfeasible in critically ill or immobile patients Contraindicated in renal dysfunction or contrast allergy
V/Q scan [19]	Useful alternative when CTPA is contraindicated Non-invasive	Limited sensitivity/specificity Affected by pre-existing lung disease It may be hard for some patients to complete
Compression Ultrasonography (CUS) [14]	Detects DVT, which can be a precursor to PE Non-invasive and accessible	Cannot confirm PE directly Only detects peripheral thrombi, not central emboli
Echocardiography [14]	Assesses right heart strain Bedside use in unstable patients Troponin/BNP can aid interpretation	Indirect evidence of PE Findings are not specific

Abbreviations: AIS, Acute Ischaemic Stroke; BNP, B-natriuretic Peptide; CUS, Compression Ultrasonography; CTPA, Computed Tomography Pulmonary Angiography; DVT, Deep Vein Thrombosis; PE, Pulmonary Embolism; and V/Q, Ventilation Perfusion.

**Table 2:** Management guidelines for VTE in AIS patients.

Guidelines	Pharmacological Prophylaxis	Mechanical Prophylaxis
American Health Association (2019) [128]	Aspirin + hydration for VTE prophylaxis in immobile stroke patients. UFH/LMWH evidence is limited, and with increased bleeding risks noted.	IPC effective (CLOTS 3 trial). GCS contraindicated.
European Stroke Organisation (2016) [27]	LMWH/UFH can be used if the VTE risk outweighs the bleeding risk. LMWH is preferred over UFH (better DVT reduction, more convenient).	Strongest evidence for IPC. GCS contraindicated. Neuromuscular electrical stimulation (NMES) needs more evidence as prophylaxis.
Neurocritical Care Society (2016) [7]	PREVAIL trial: LMWH superior to UFH for DVT prevention in AIS. Pharmacological and mechanical prophylaxis may act synergistically.	CLOTS 3 trial: IPC = absolute risk reduction of 3.6% of VTE when started within 0–3 days post-stroke. GCS may dislodge VTE and cause skin breakdown in immobile patients.
European Society of Cardiology (2021) [129]	Limited specific recommendations on PE prophylaxis in AIS patients. Early anticoagulation (<48 h) after AIS can increase the risk of intracranial haemorrhage.	No clear recommendation.
Canadian Stroke Best Practice (2022) [130]	LMWH/UFH on admission if no contraindications (e.g., haemorrhage).	IPC recommended. GCS not recommended.

Abbreviations: AIS, Acute Ischaemic Stroke; CLOTS 3, Clots in Legs Or sTockings after Stroke; GCS, Graduated Compression Stockings; IPC, Intermittent Pneumatic Compression; LMWH, Low-Molecular-Weight Heparin; NMES, Neuromuscular Electrical Stimulation; PE, Pulmonary Embolism; UFH, Unfractionated Heparin; and VTE, Venous Thromboembolism.

## Data Availability

The original contributions presented in this study are included in the article. Further inquiries can be directed to the corresponding author.
